# Investigation of electrophysiological markers to predict clinical and functional outcome of schizophrenia using sparse partial least square regression

**DOI:** 10.1192/j.eurpsy.2021.1446

**Published:** 2021-08-13

**Authors:** L. Giuliani, D. Popovic, N. Koutsouleris, G.M. Giordano, T. Koenig, A. Mucci, A. Vignapiano, M. Altamura, A. Bellomo, R. Brugnoli, G. Corrivetti, G. Di Lorenzo, P. Girardi, P. Monteleone, C. Niolu, S. Galderisi, M. Maj

**Affiliations:** 1 Psychiatry, Università degli Studi della Campania “Luigi Vanvitelli”, Napoli, Italy; 2 Department Of Psychiatry And Psychotherapy, LMU Munich, Munich, Germany; 3 Psychiatry, University Hospital of Psychiatry, Bern, Bern, Switzerland; 4 Psichiatria, ASL Napoli 1 - Centro, Napoli, Italy; 5 Psychiatry, University of Foggia, Foggia, Italy; 6 Psychiatry, University of Rome “La Sapienza”, Rome, Italy; 7 Department Of Psychiatry, 10European Biomedical Research Institute of Salerno (EBRIS), salerno, Italy; 8 Psychiatry, University of Rome “Tor Vergata”, Rome, Italy; 9 Department Of Medicine, Surgery And Dentistry “scuola Medica Salernitana”, University of Salerno, Baronissi/Salerno, Italy; 10 Department Of Systems Medicine, University of Rome “Tor Vergata”, Roma, Italy

**Keywords:** schizophrénia, EEG, Outcome prediction

## Abstract

**Introduction:**

Despite innovative treatments, the impairment in real-life functioning in subjects with schizophrenia (SCZ) remains an unmet need in the care of these patients. Recently, real-life functioning in SCZ was associated with abnormalities in different electrophysiological indices. It is still not clear whether this relationship is mediated by other variables, and how the combination of different EEG abnormalities influences the complex outcome of schizophrenia.

**Objectives:**

The purpose of the study was to find EEG patterns which can predict the outcome of schizophrenia and identify recovered patients.

**Methods:**

Illness-related and functioning-related variables were measured in 61 SCZ at baseline and after four-years follow-up. EEGs were recorded at the baseline in resting-state condition and during two auditory tasks. We performed Sparse Partial Least Square (SPLS) Regression, using EEG features, age and illness duration to predict clinical and functional features at baseline and follow up. Through a Linear Support Vector Machine (Linear SVM) we used electrophysiological and clinical scores derived from SPLS regression, in order to classify recovered patients at follow-up.

**Results:**

We found one significant latent variable (p<0.01) capturing correlations between independent and dependent variables at follow-up (RHO=0.56). Among individual predictors, age and illness-duration showed the highest scores; however, the score for the combination of the EEG features was higher than all other predictors. Within dependent variables, negative symptoms showed the strongest correlation with predictors. Scores resulting from SPLS Regression classified recovered patients with 90.1% of accuracy.

**Conclusions:**

A combination of electrophysiological markers, age and illness-duration might predict clinical and functional outcome of schizophrenia after 4 years of follow-up.

